# Exogenous Gibberellins and Auxins Promote Crown Bud Regeneration and Influence Endogenous Hormone Changes in Alfalfa

**DOI:** 10.3390/plants14111699

**Published:** 2025-06-02

**Authors:** Haiyan Yue, Qunce Sun, Shuzhen Zhang, Youping An, Xianwei Peng, Binghan Wen, Xingyu Ge, Yuxiang Wang

**Affiliations:** 1 College of Grassland Science, Xinjiang Agricultural University, Urumqi 830052, China; hy713812@163.com (H.Y.); 18678637275@163.com (Q.S.); 18399820280@163.com (Y.A.); psuyy521@163.com (X.P.); 15862260175@163.com (B.W.); 18255347015@163.com (X.G.); wyx9868@163.com (Y.W.); 2 Xinjiang Key Laboratory of Grassland Resources and Ecology, Urumqi 830052, China; 3 The Ministry of Education Key Laboratory of Grassland Resources and Ecology in the Western Arid and Desert Areas, Urumqi 830052, China

**Keywords:** exogenous growth regulators, alfalfa, crown buds, regeneration, endogenous hormones

## Abstract

Alfalfa (*Medicago sativa* L.) is a globally significant forage crop with notable economic value. Gibberellins (GA_3_) promote dormancy breaking and early germination whereas auxins (IAA) predominantly influence bud regeneration. This study investigated the effects of exogenous gibberellins and indole acetic acid on the regeneration and biomass of crown buds in two alfalfa varieties with different dormancy levels. The experiment involved five concentrations each of gibberellins (0 mg/L, 10 mg/L, 20 mg/L, 30 mg/L, and 40 mg/L) and auxins (0 mg/L, 5 mg/L, 10 mg/L, 15 mg/L, and 20 mg/L). The results indicated that both exogenous gibberellins and auxins significantly increased the endogenous levels of these hormones in the crown buds, while decreasing abscisic acid (ABA) levels. There was also a significant increase in sugar and total nitrogen content in the buds. Treatments with exogenous gibberellins enhanced the number of crown buds and the aboveground biomass per plant, with the best results at 30 mg/L. Auxin treatments showed the largest increase in aboveground biomass per plant at 15 mg/L. In summary, 30 mg/L gibberellins or 15 mg/L auxins is recommended as the optimal spraying concentration. This research provides practical evidence for the regulation of exogenous growth regulators in alfalfa cultivation.

## 1. Introduction

Alfalfa (*Medicago sativa* L.) is the most widely cultivated perennial leguminous forage in the world today, known for its high nutritional value, cutting tolerance, high yield, and strong adaptability [1,2]. Fall dormancy (FD) is a growth trait of alfalfa, where in mid- to high-latitude regions of the Northern Hemisphere, reduced autumn sunlight and lower temperatures lead to changes in the morphological types and productive performance of alfalfa. Fall dormancy plays a crucial role in the evaluation of alfalfa’s cold tolerance and in breeding selection [3]. Varieties with different levels of fall dormancy exhibit various agronomic traits [4]. Research has found that the annual yield of alfalfa decreases with reduced dormancy [5]. Non-dormant varieties flower earlier than dormant types, have higher autumn yields, and can quickly regreen with timely spring irrigation, with faster regrowth after summer cutting [6]. In both summer and autumn, non-dormant alfalfa varieties exhibit faster leaf area expansion rates, plant growth speeds, and biological yields after cutting compared to dormant varieties [7]. Therefore, enhancing the productive performance of low-dormancy alfalfa is of significant importance for forage production.

Crown bud development capacity is an important potential selection criterion for improving alfalfa yield [8]. Regrowth after cutting alfalfa significantly affects its annual and seasonal yield, as well as plant persistence, with new shoots during the regeneration period, especially during the spring green-up, primarily arising from crown buds [9]. An appropriate environment for crown bud germination is one of the key factors in enhancing alfalfa yield [10]. Alfalfa genotypes with multiple crown buds mature earlier and thrive at lower optimal temperatures compared to those with fewer buds [11], and they are characterized by high quality and yield, as well as resistance to disease, drought, and cold [12]. Regrowth branches developed from crown buds after cutting alfalfa dominate the contribution to forage yield [13]. Previous studies have shown that the regeneration speed of axillary buds is slower than that of crown buds, and the branches developed from crown buds attain a larger biomass, contributing up to about two thirds of the total forage yield [14,15].

**Figure 1:**
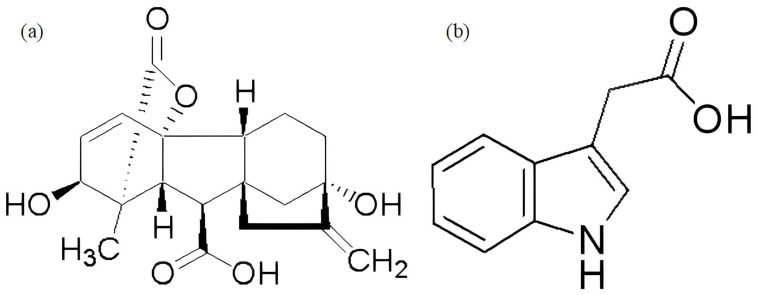
(**a**) shows the chemical structure of gibberellin (GA_3_) and (**b**) shows the chemical structure of auxin (3-indoleacetic acid, IAA).

Plant endogenous hormones are highly active substances within plants, participating in and regulating many physiological processes and growth development. Although present in minute amounts, they play a significant role in plant regulation [16,17]. Currently, commonly used growth regulators include gibberellins (GAs), paclobutrazol (PP333), auxins (IAA), and abscisic acid (ABA), among others [18,19]. Most studies have found that plant growth regulators can significantly improve the rooting rate of cuttings propagation [20,21]. Exogenous gibberellic acid (GA_3_) has long been used to break dormancy and promote the growth of roots, stems, and leaves [22,23]; its basic structural formula is shown in Figure 1a. Indole-3-acetic acid (IAA) is a plant growth hormone that plays a key role in plant cell division, growth, and differentiation [24]; its basic structural formula is shown in Figure 1b. The endogenous hormone IAA is important in the induction and expression of adventitious roots and the formation of callus tissue [25]. Additionally, nitric oxide (NO) is also involved in auxin-induced lateral root formation [26]. Remodeling of roots exposed to salinity is coordinated by complex interactions among various signaling pathways involving phytohormones, nitric oxide (NO), and reactive oxygen species (ROS). Polyamines (PAs), small, cationic amine molecules with diverse roles in plant growth and stress responses, are also known to influence root morphology [27]. The simultaneous application of AsV and Si up-regulated the expression of genes involved in nitric oxide (NO) metabolism, cell cycle progression, auxin (IAA, indole-3-acetic acid) biosynthesis and transport, and Si uptake, which accompanied adventitious root formation [28]. An important regulator during root development and regeneration is the gasotransmitter nitric oxide (NO) [29]. Amandeep Kaur and others, with pecan as their research subject, reviewed the transcriptomic analysis of plant hormones and the role of exogenous application in pecan bud fissure, flowering, and fruiting. They found that different plant hormones (auxins, GAs, cytokinins (CTK), ABA, ethylene, salicylic acid (SA), and jasmonic acid (JA)) have varying levels of importance in the flowering process [30]. Additionally, plant growth regulators play a crucial role in regulating crop leaf senescence. Exogenous GA_3_ treatment promotes rapid early growth in rice plants, resulting in taller plants and larger leaf expansion. IAA treatment delays leaf senescence and reduces yield loss by enhancing crop growth rate, leaf function, and root activity and increasing the contribution of nonstructural carbohydrates (NSC) from the stem to the grains [31]. Wen-hui Xie and others, for the first time, sprayed different concentrations of growth regulators (indole-3-acetic acid, IAA, and gibberellic acid A_3_, GA_3_) on dwarf mutant shrubs of high-quality forage Senna and found significant differences in the response of dwarf mutant plants to exogenous IAA and GA_3_ in terms of endogenous hormone content and plant height changes [32]. Song Jiaqi [33] found that treatment with 20 mg/L of 6-BA promotes the synthesis of IAA, GA_3_, and CTK in alfalfa leaves and inhibits the synthesis of ABA. Li Hong [34] found that exogenous ABA plays an important role in regulating the changes in the content of IAA, GA_3_, and ABA hormones in the roots and leaves of alfalfa seedlings under soda saline–alkali stress, contributing to alfalfa’s salt–alkali tolerance.

Currently, research on the application of exogenous growth regulators to alfalfa to increase yield mainly focuses on seed soaking and leaf spraying. The effects of different exogenous growth regulators on the regeneration of crown buds and yield increase in alfalfas with different levels of autumn dormancy, as well as the mechanisms of action of various hormones, still need further exploration. Therefore, this experiment selected two types of alfalfa with different levels of autumn dormancy as experimental materials. Two exogenous growth regulators, GA_3_ and IAA, are set with progressively increasing concentration levels. The dynamic changes in the content of endogenous hormones, soluble sugars, starch, and total nitrogen in the crown buds of alfalfa under different treatments are measured, and the effects of exogenous GA_3_ and IAA spraying on the regeneration and biomass of alfalfa crown buds are clarified, in order to provide a theoretical basis for enhancing the production performance of low-fall-dormancy-level alfalfa.

## 2. Results

### 2.1. Effects of Exogenous GA_3_ and IAA Spraying on the Regeneration and Biomass of Alfalfa Crown Buds

#### 2.1.1. Effects of Exogenous GA_3_ and IAA Spraying on the Regeneration of Alfalfa Crown Buds

**Figure 2:**
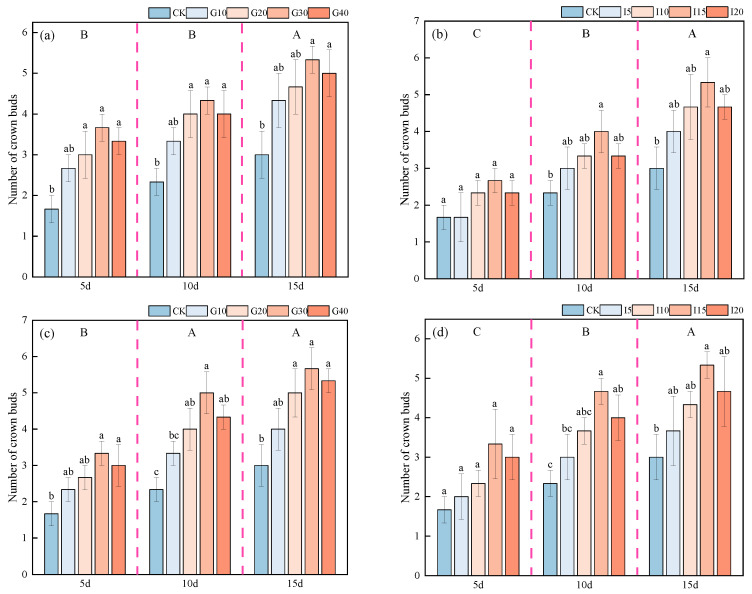
Effects of exogenous GA_3_ and IAA spraying on the number of crown buds in ‘Xinmu No. 4’ (**a**,**b**) and ‘Saiwo’ (**c**,**d**) alfalfa. In the figure, different lowercase letters indicate significant differences between treatments with different spraying concentrations at the same time point (*p* < 0.05); different uppercase letters indicate significant differences between different observation periods (5 d, 10 d, 15 d) (*p* < 0.05).

The effects of exogenous GA_3_ and IAA spraying on the regeneration of crown buds in ‘Xinmu No. 4’ and ‘Saiwo’ alfalfa are shown in Figure 2. The number of crown buds in both ‘Xinmu No. 4’ and ‘Saiwo’ alfalfa showed a significant increasing trend over time with the application of GA_3_ and IAA, reaching the highest 15 days post-treatment (*p* < 0.05). Under the treatment with the two exogenous growth regulators, GA_3_ and IAA, the number of crown buds in both types of alfalfa initially increased and then decreased with rising concentrations. Specifically, under the GA_3_ treatment, the G30-level alfalfa showed the highest number of crown buds across all three observation periods, significantly higher than the control (CK) alfalfa (*p* < 0.05), with increases of 77.78% and 88.89%, respectively. Under the IAA treatment, the I15-level alfalfa showed the highest number of crown buds across all periods, significantly higher than the control (CK) alfalfa (*p* < 0.05), with an increase of 77.78% in both types of alfalfa.

#### 2.1.2. The Effect of Exogenous GA_3_ and IAA Spraying on the Length of Crown Buds in Alfalfa

**Figure 3:**
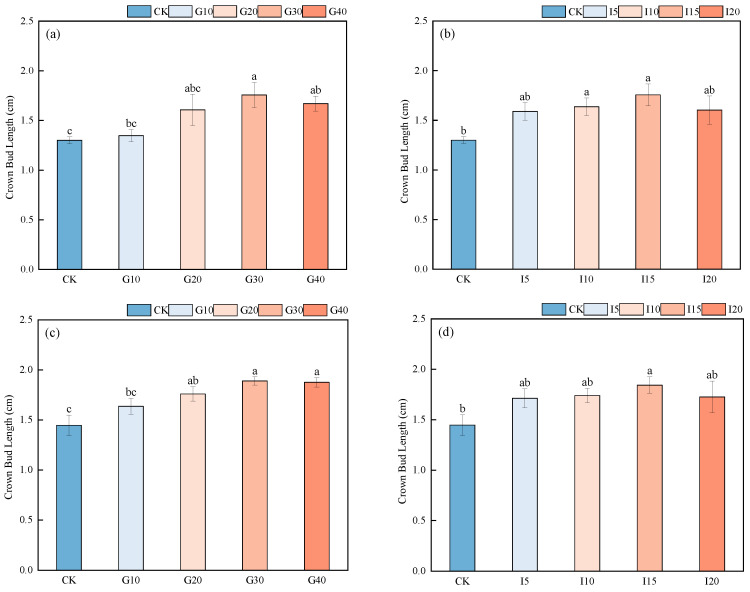
The effect of exogenous GA_3_ and IAA spraying on the length of crown buds in alfalfa varieties “Xinmu No. 4” (**a**,**b**) and “Saiwo” (**c**,**d**). Different lowercase letters in the figure indicate significant differences between treatments (*p* < 0.05).

As shown in Figure 3, for the new pasture variety No. 4, there was no significant difference in the average length of crown buds among different GA_3_ concentration treatments (*p* > 0.05). After different concentrations of IAA spraying, the average length of crown buds with the I15 treatment was significantly higher than that with the control (CK) treatment (*p* < 0.05), with the average crown bud length ranking as I15 > I10 > I20 > I5 > CK. For the variety Saiwo, after spraying with different concentrations of GA_3_, the average length of crown buds for G30 was significantly higher than that for CK (*p* < 0.05), with lengths ranking as G30 > G40 > G20 > G10 > CK; after different concentrations of IAA spraying, the I15 treatment alfalfa also showed a significantly higher average length than the CK alfalfa (*p* < 0.05), with lengths ranking as I15 > I10 > I20 > I5 > CK.

#### 2.1.3. The Effect of Exogenous GA_3_ and IAA Spraying on the Neck Traits of Alfalfa Roots

**Figure 4:**
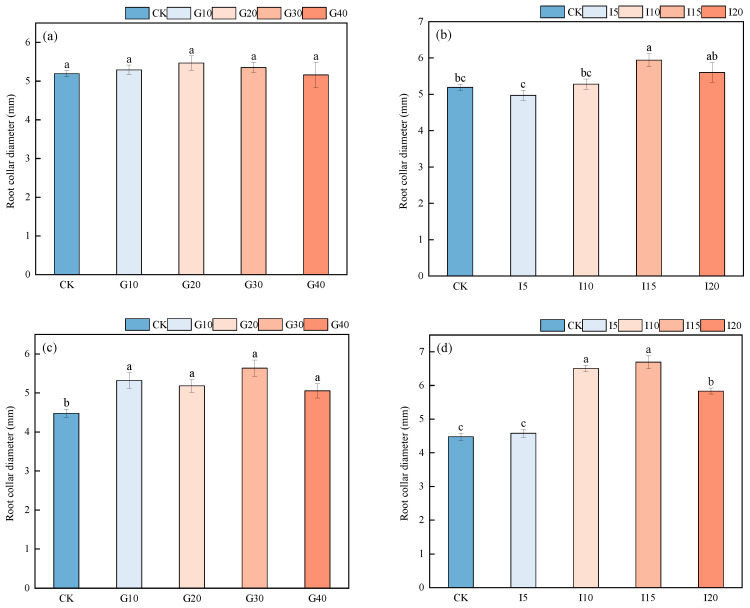
The effect of exogenous GA_3_ and IAA spraying on the neck diameter of alfalfa varieties “Xinmu No. 4” (**a**,**b**) and “Saiwo” (**c**,**d**). Different lowercase letters indicate significant differences between treatments (*p* < 0.05).

As shown in Figure 4, for the variety “Xinmu No. 4”, there was no significant difference in average neck diameter after spraying with different concentrations of GA_3_ (*p* > 0.05). After different concentrations of IAA spraying, the I15 treatment significantly outperformed other groups (*p* < 0.05), with the average neck diameter ranking as I15 > I20 > I10 > CK > I5. For the variety “Saiwo”, after different concentrations of GA_3_ spraying, the average neck diameter for the G30 alfalfa was significantly longer than it was for the CK alfalfa (*p* < 0.05), but there was no significant difference between G30 and G10, G20, or G40 treatments (*p* > 0.05). After different concentrations of IAA spraying, the I15-treatment alfalfa had a significantly longer average neck diameter than the CK, I5, and I20 alfalfa (*p* < 0.05), but it was not significantly different from that of the I10 alfalfa (*p* > 0.05).

**Figure 5:**
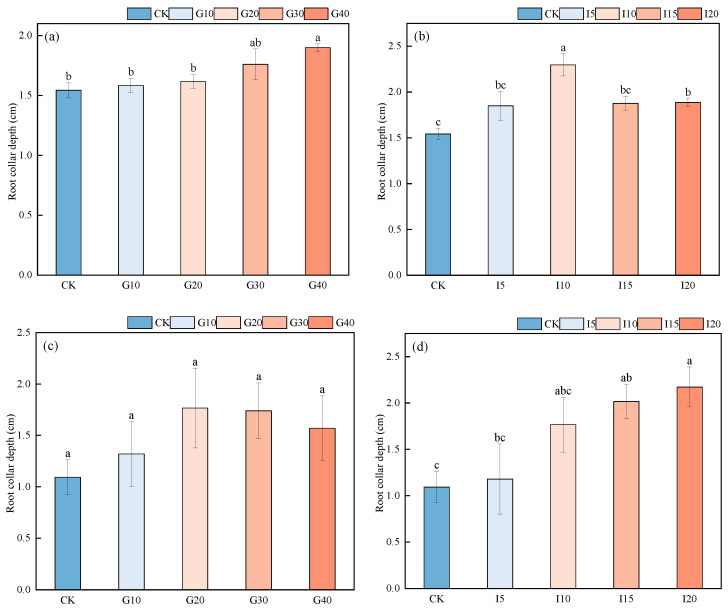
The effect of exogenous GA_3_ and IAA spraying on the depth of neck penetration into the soil for alfalfa varieties “Xinmu No. 4” (**a**,**b**) and “Saiwo” (**c**,**d**). Different lowercase letters indicate significant differences between treatments (*p* < 0.05). This figure visually represents the statistical outcomes of the experimental treatments on neck penetration depth.

As shown in Figure 5, for “Xinmu No. 4”, after different concentrations of GA_3_ spraying, G40 alfalfa showed a significantly greater average depth of neck penetration into the soil than CK, G10, and G20 alfalfa (*p* < 0.05). After different concentrations of IAA spraying, the I10 treatment significantly outperformed the other groups in the depth of neck penetration (*p* < 0.05), ranking as I10 > I20 > I15 > I5 > CK. For “Saiwo”, there was no significant difference in the average depth of neck penetration after GA_3_ treatments (*p* > 0.05); however, after IAA treatments, the I20 treatment alfalfa showed a significantly longer neck diameter than CK (*p* < 0.05), with depths ranking as I20 > I15 > I10 > I5 > CK.

**Figure 6:**
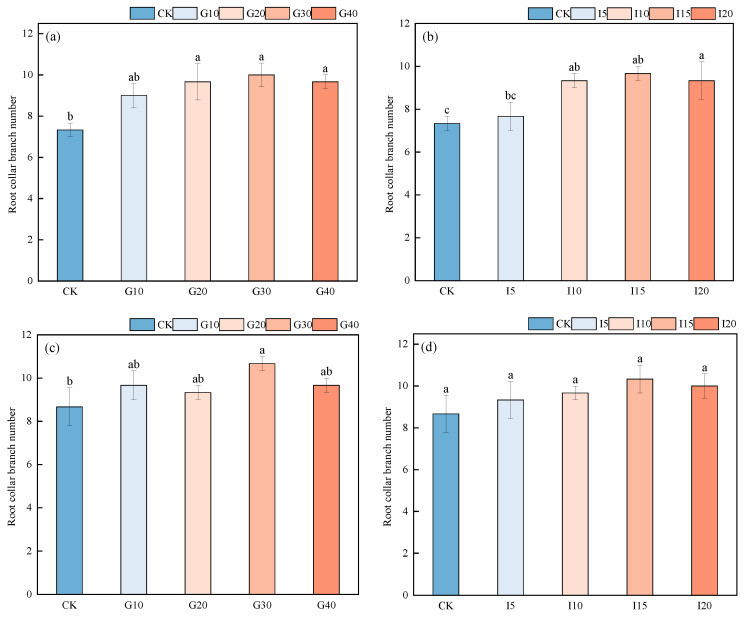
The effect of exogenous GA_3_ and IAA spraying on the number of crown branches in alfalfa varieties “Xinmu No. 4” (**a**,**b**) and “Saiwo” (**c**,**d**). Different lowercase letters in the figure indicate significant differences between treatments (*p* < 0.05).

As shown in Figure 6, for “Xinmu No. 4”, there was no significant difference in the average number of neck branches after spraying with GA_3_ at concentrations of G20, G30, and G40 (*p* > 0.05). After IAA spraying, the I15 treatment significantly outperformed CK (*p* < 0.05), with the average number of neck branches ranking as I15 > I10 > I20 > I5 > CK. For “Saiwo”, after GA_3_ spraying, the average number of neck branches for G30 was significantly higher than it was for CK (*p* < 0.05), ranking as G30 > G40 = G10 > G20 > CK. After IAA spraying, the I15 treatment alfalfa had significantly more neck branches than the CK alfalfa (*p* < 0.05), with the ranking as I15 > I20 > I10 > I5 > CK.

#### 2.1.4. Effects of Exogenous GA_3_ and IAA Spraying on Aboveground Biomass of Alfalfa

**Figure 7:**
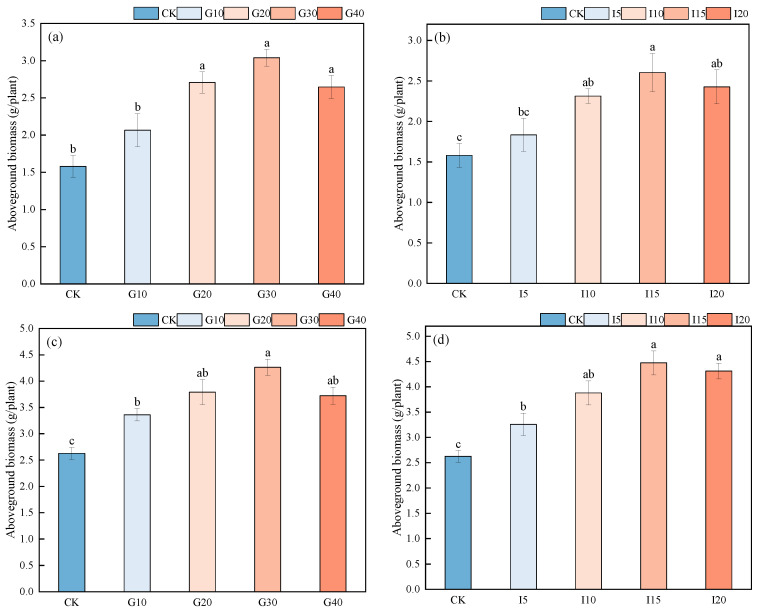
Effects of Exogenous GA_3_ and IAA on the aboveground biomass of ‘Xinmu No. 4’ (**a**,**b**) and ‘Saiwo’ (**c**,**d**) alfalfa. Different lowercase letters in the figure indicate significant differences between treatments (*p* < 0.05).

The impact of exogenous GA_3_ and IAA on the aboveground biomass of ‘Xinmu No. 4’ and ‘Saiwo’ alfalfa is illustrated in Figure 7. For ‘Xinmu No. 4’, after spraying with different concentrations of GA_3_, there were no significant differences among G20, G30, and G40 (*p* > 0.05). Compared to the control (CK), the average increases in aboveground biomass for G10, G20, G30, and G40 were 30.80%, 71.31%, 92.41%, and 67.51%, respectively. After spraying with different concentrations of IAA, compared to CK, the average increases in aboveground biomass for I5, I10, I15, and I20 were 16.03%, 46.41%, 64.77%, and 53.29%, respectively. For ‘Saiwo’, after application of different concentrations of GA_3_, the average increases in aboveground biomass compared to CK for G10, G20, G30, and G40 were 27.92%, 44.29%, 62.31%, and 41.75%, respectively. After spraying with different concentrations of IAA, compared to CK, the average increases in aboveground biomass for I5, I10, I15, and I20 were 23.98%, 47.72%, 70.30%, and 64.21%, respectively.

### 2.2. Effects of Exogenous GA_3_ and IAA on Endogenous GA_3_, IAA, and ABA Content in Alfalfa Crown Buds

#### 2.2.1. Effects of Exogenous GA_3_ and IAA on Endogenous GA_3_ Content in Alfalfa Crown Buds

**Figure 8:**
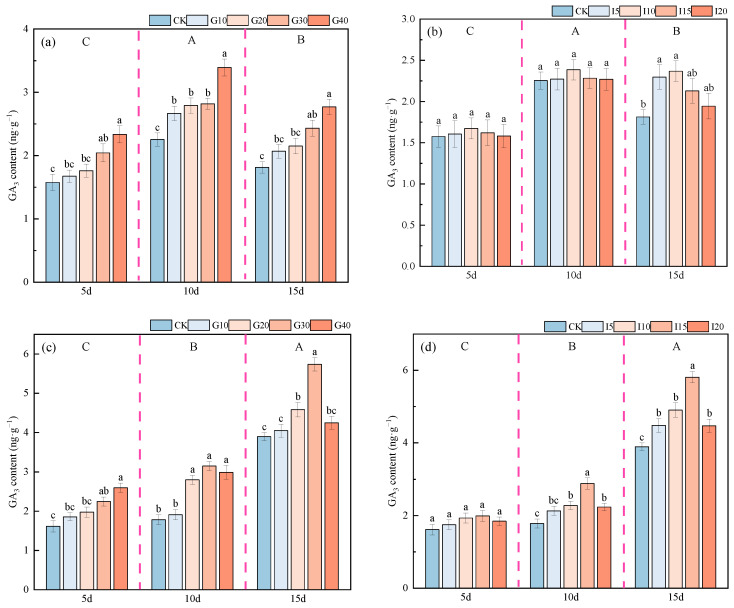
Effects of Exogenous GA_3_ and IAA on endogenous GA_3_ content in the crown buds of ‘Xinmu No. 4’ (**a**,**b**) and ‘Saiwo’ (**c**,**d**) alfalfa. Different lowercase letters in the figure indicate significant differences between treatments within a certain period (*p* < 0.05); different uppercase letters indicate significant differences between different periods (*p* < 0.05).

As shown in Figure 8, for the variety “Xinmu No. 4” alfalfa, the endogenous GA_3_ content in the crown buds reached its highest level 10 days after spraying, significantly higher than at other times (*p* < 0.05); for “Saiwo” alfalfa, the peak was reached 15 days post-spray, also significantly higher than other periods (*p* < 0.05). For “Xinmu No. 4”, under exogenous GA_3_ application, the endogenous GA_3_ content in the crown buds increased with higher concentrations, with the G40 level being the highest across all three observation periods. Under IAA treatment, the trend was initially increasing and then decreasing, with the I10 level being the highest across all observation periods, significantly higher than the control (CK) (*p* < 0.05). For “Saiwo”, under both GA_3_ and IAA treatments, the endogenous GA_3_ content showed a trend of initially increasing and then decreasing, with the highest level during the third observation period under G30 for GA_3_ treatment, and the highest level across all periods under I15 for IAA treatment, significantly higher than CK (*p* < 0.05).

#### 2.2.2. Effects of Exogenous GA_3_ and IAA on Endogenous IAA Content in Alfalfa Crown Buds

**Figure 9:**
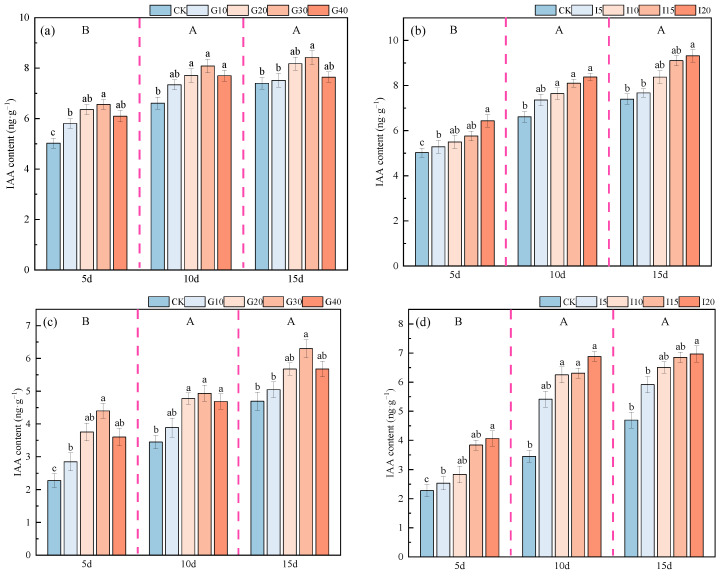
Effects of exogenous GA_3_ and IAA on endogenous IAA content in the crown buds of ‘Xinmu No. 4’ (**a**,**b**) and ‘Saiwo’ (**c**,**d**) alfalfa. Different lowercase letters in the figure indicate significant differences between treatments within a certain period (*p* < 0.05); different uppercase letters indicate significant differences between different periods (*p* < 0.05).

The impact of exogenous GA_3_ and IAA on the endogenous IAA content in the crown buds of ‘Xinmu No. 4’ and ‘Saiwo’ alfalfa is illustrated in Figure 9. For both varieties, the endogenous IAA content showed an increasing trend over time following the application of exogenous GA_3_ and IAA, reaching the highest level after 15 days, significantly higher than at other times (*p* < 0.05). For ‘Xinmu No. 4’, under exogenous GA_3_ treatment, the endogenous IAA content initially increased and then decreased with increasing concentrations, with the highest content at the G30 level across all three observation periods. Under exogenous IAA treatment, the endogenous IAA content consistently increased with concentration, with the highest content at the I20 level across all observation periods, significantly higher than that of the control (CK) (*p* < 0.05). For ‘Saiwo’, under exogenous GA_3_ treatment, the endogenous IAA content also showed an initial increase followed by a decrease with increasing concentrations, with the highest content at the G30 level during the third observation period. Under exogenous IAA treatment, the endogenous IAA content consistently increased with concentration, with the highest content at the I20 level across all three observation periods, significantly higher than that of CK (*p* < 0.05).

#### 2.2.3. Effects of Exogenous GA_3_ and IAA on Endogenous ABA Content in Alfalfa Crown Buds

**Figure 10:**
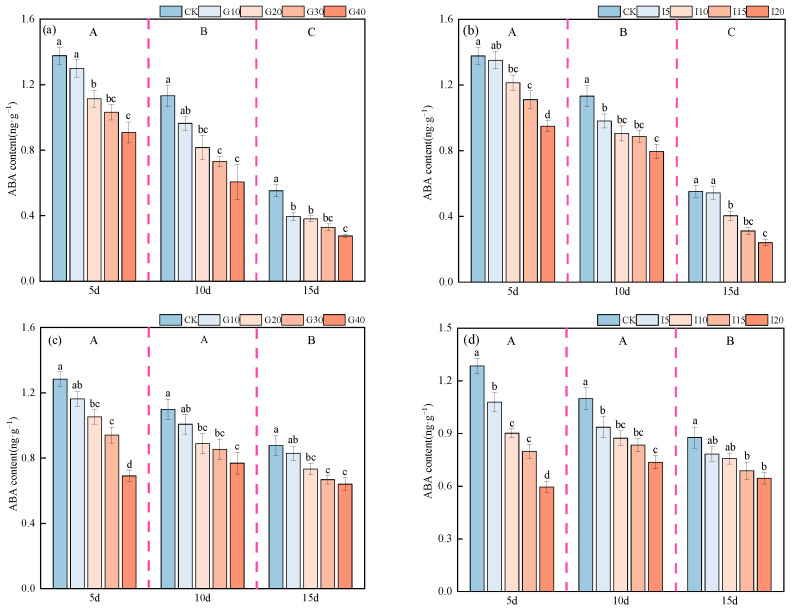
Effects of exogenous GA_3_ and IAA on endogenous ABA content in the crown buds of ‘Xinmu No. 4’ (**a**,**b**) and ‘Saiwo’ (**c**,**d**) alfalfa. Different lowercase letters in the figure indicate significant differences between treatments within a certain period (*p* < 0.05); different uppercase letters indicate significant differences between different periods (*p* < 0.05).

The impact of exogenous GA_3_ and IAA on the endogenous ABA content in the crown buds of ‘Xinmu No. 4’ and ‘Saiwo’ alfalfa is illustrated in Figure 10. For both varieties, the endogenous ABA content showed a decreasing trend over time following the application of exogenous GA_3_ and IAA, reaching the highest level after 5 days, significantly higher than at other times (*p* < 0.05).For both ‘Xinmu No. 4’ and ‘Saiwo’, under the treatment with exogenous growth regulators GA_3_ and IAA, the endogenous ABA content in the crown buds decreased with increasing concentrations. The control (CK) level of endogenous ABA content was the highest across all three observation periods, significantly higher than it was for other treatments (*p* < 0.05).

#### 2.2.4. Effects of Exogenous GA_3_ and IAA on the Ratio of Endogenous GA_3_ to ABA in Alfalfa Crown Buds

**Figure 11:**
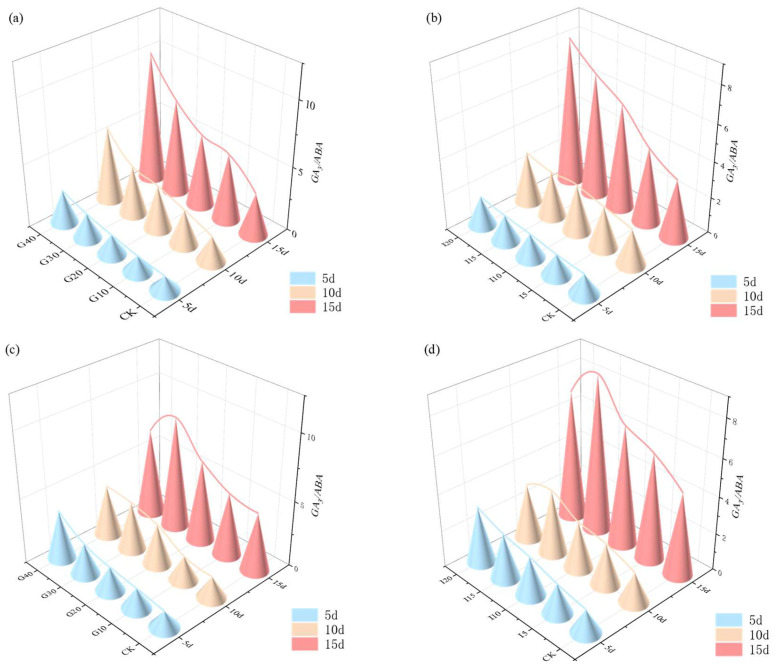
Effects of exogenous GA_3_ and IAA on the ratio of endogenous GA_3_/ABA in the crown buds of ‘Xinmu No. 4’ (**a**,**b**) and ‘Saiwo’ (**c**,**d**) alfalfa.

The impact of exogenous GA_3_ and IAA on the ratio of endogenous GA_3_ to ABA in the crown buds of ‘Xinmu No. 4’ and ‘Saiwo’ alfalfa is shown in Figure 11. For ‘Xinmu No. 4’, under different concentrations of exogenous GA_3_ and IAA, the average endogenous GA_3_/ABA ratio in the crown buds increased with the hormone concentration, reaching the highest at 15 days with the G40 treatment. For ‘Saiwo’, the average endogenous GA_3_/ABA ratio in the crown buds initially increased and then decreased with rising hormone concentrations, with the highest ratio achieved at 15 days under the I15 treatment.

#### 2.2.5. Effects of Exogenous GA_3_ and IAA on the Ratio of Endogenous IAA to ABA in Alfalfa Crown Buds

**Figure 12:**
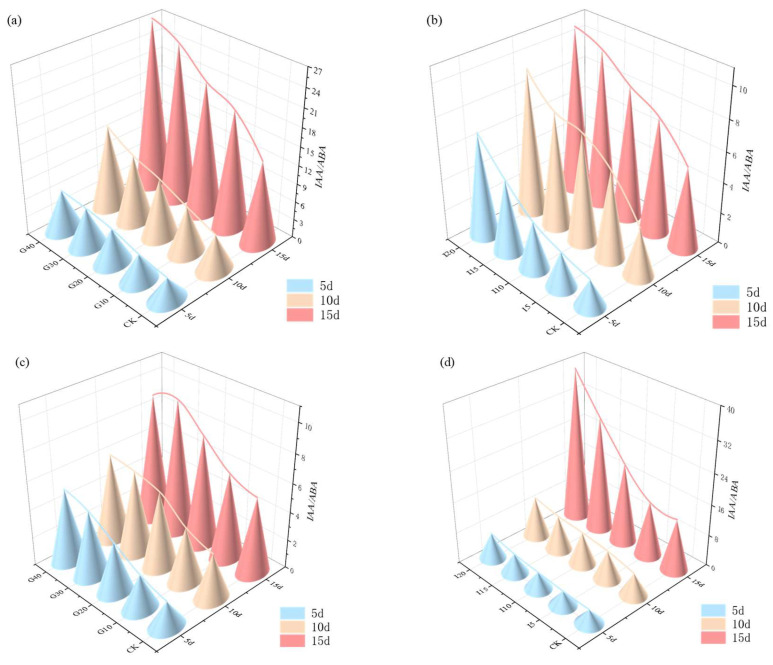
Effects of exogenous GA_3_ and IAA on the ratio of endogenous IAA/ABA in the crown buds of ‘Xinmu No. 4’ (**a**,**b**) and ‘Saiwo’ (**c**,**d**) alfalfa.

The impact of exogenous GA_3_ and IAA on the ratio of endogenous IAA to ABA in the crown buds of ‘Xinmu No. 4’ and ‘Saiwo’ alfalfa is illustrated in Figure 12. For ‘Xinmu No. 4’, under different concentrations of exogenous GA_3_ and IAA, the average endogenous IAA/ABA ratio in the crown buds increased with the hormone concentration, reaching the highest level at 15 days with the G40 treatment. For ‘Saiwo’, the average endogenous IAA/ABA ratio in the crown buds initially increased and then decreased with rising hormone concentrations, reaching the highest level at 15 days under the I15 treatment.

### 2.3. Effects of Exogenous GA_3_ and IAA on Soluble Sugar, Starch, and NSC Content in Alfalfa Crown Buds

#### 2.3.1. Effects of Exogenous GA_3_ and IAA on Soluble Sugar Content in Alfalfa Crown Buds

**Figure 13:**
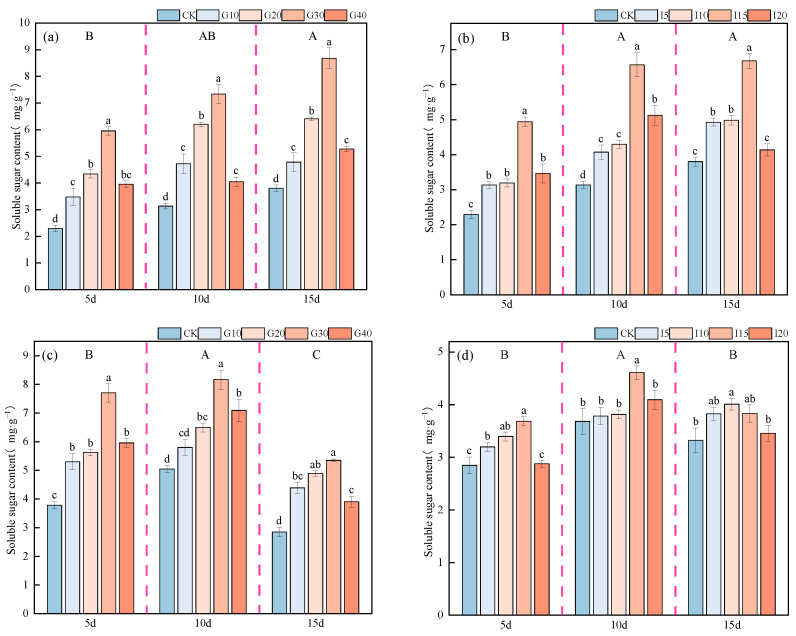
Effects of exogenous GA_3_ and IAA on soluble sugar content in the crown buds of ‘Xinmu No. 4’ (**a**,**b**) and ‘Saiwo’ (**c**,**d**) alfalfa. Different lowercase letters in the figure indicate significant differences between treatments within a certain period (*p* < 0.05); different uppercase letters indicate significant differences between different periods (*p* < 0.05).

As shown in Figure 13, the soluble sugar content in the crown buds of “Xinmu No. 4” alfalfa reached its highest level 15 days after spraying, significantly higher than at other times (*p* < 0.05); for “Saiwo” alfalfa, the peak was reached 10 days post-spray, also significantly higher than other periods (*p* < 0.05). For “Xinmu No. 4”, under exogenous GA_3_ application, the soluble sugar content in the crown buds initially increased and then decreased with rising concentrations, with the G30 level being the highest across all three observation periods. Under IAA treatment, the soluble sugar content consistently increased with concentration, with that at the I15 level being the highest across all observation periods, significantly higher than that for the control (CK) (*p* < 0.05). For “Saiwo”, under GA_3_ treatment, the soluble sugar content showed a trend of initially increasing and then decreasing, with the highest level during the third observation period at G30. Under IAA treatment, the trend was consistently increasing, with the soluble sugar content at the I15 level being the highest in two observation periods, significantly higher than that for CK (*p* < 0.05).

#### 2.3.2. Effects of Exogenous GA_3_ and IAA on Starch Content in Alfalfa Crown Buds

**Figure 14:**
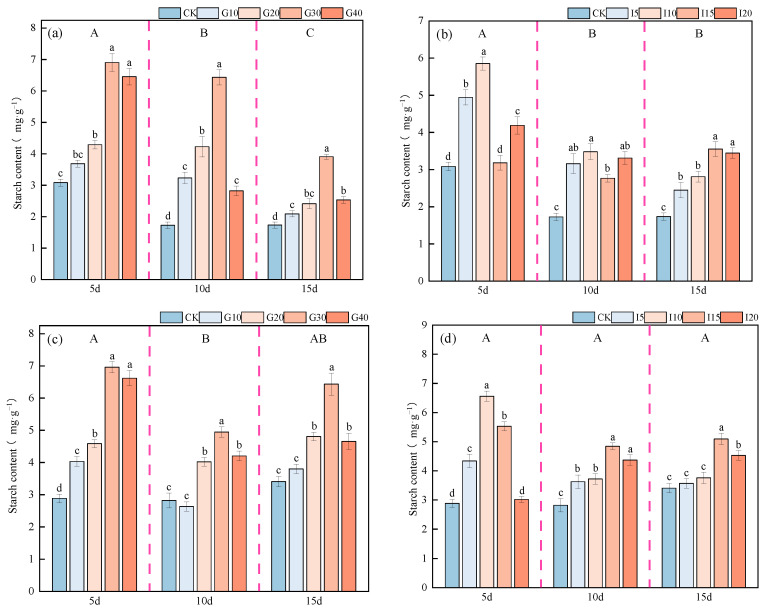
Effects of exogenous GA_3_ and IAA on starch content in the crown buds of ‘Xinmu No. 4’ (**a**,**b**) and ‘Saiwo’ (**c**,**d**) alfalfa. Different lowercase letters in the figure indicate significant differences between treatments within a certain period (*p* < 0.05); different uppercase letters indicate significant differences between different periods (*p* < 0.05).

The impact of exogenous GA_3_ and IAA on the starch content in the crown buds of ‘Xinmu No. 4’ and ‘Saiwo’ alfalfa is shown in Figure 14. For ‘Xinmu No. 4’, the starch content in the crown buds exhibited a decreasing trend over time after spraying with exogenous GA_3_ and IAA, with the highest level observed after 5 days, significantly higher than at other times (*p* < 0.05). For ‘Saiwo’, the starch content in the crown buds initially decreased and then increased over time after spraying with exogenous GA_3_, with the highest level observed after 5 days, significantly higher than at other times (*p* < 0.05). For ‘Xinmu No. 4’ alfalfa, under the treatment with exogenous GA_3_, the starch content in the crown buds first increased and then decreased with increasing concentrations, with the highest content at the G30 level across all three observation periods. Under exogenous IAA treatment, the starch content showed a trend of initially increasing, then decreasing, and finally increasing again with increased concentrations, with the highest content at the I10 level in the first two observation periods, significantly higher than for the control (CK) (*p* < 0.05), and at the I15 level in the third observation period, significantly higher than for CK (*p* < 0.05). For ‘Saiwo’ alfalfa, under the treatment with exogenous GA_3_ and IAA, the starch content in the crown buds showed a trend of initially increasing and then decreasing with increased concentrations. The highest starch content was at the G30 level for GA_3_ treatment across all observation periods, at the I10 level for IAA treatment during the first observation period, and at the I15 level for the subsequent two observation periods, significantly higher than for CK (*p* < 0.05).

#### 2.3.3. Effects of Exogenous GA_3_ and IAA on NSC Content in Alfalfa Crown Buds

**Figure 15:**
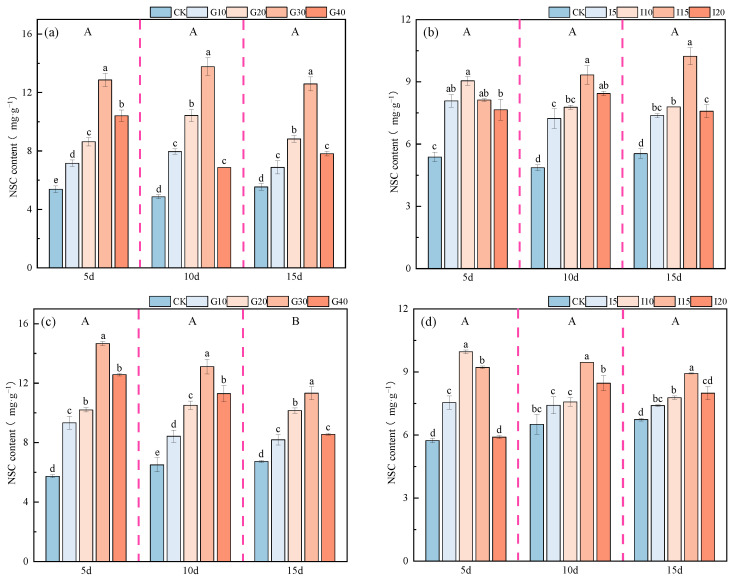
Effects of exogenous GA_3_ and IAA on NSC content in the crown buds of ‘Xinmu No. 4’ (**a**,**b**) and ‘Saiwo’ (**c**,**d**) alfalfa. Different lowercase letters indicate significant differences between treatments within a certain period (*p* < 0.05); different uppercase letters indicate significant differences between different periods (*p* < 0.05).

The impact of exogenous GA_3_ and IAA on the NSC content in the crown buds of ‘Xinmu No. 4’ and ‘Saiwo’ alfalfa is shown in Figure 15. The NSC content in the crown buds of both ‘Xinmu No. 4’ and ‘Saiwo’ alfalfa remained unchanged over time with the application of exogenous GA_3_ and IAA. For ‘Xinmu No. 4’ alfalfa, under the treatment with exogenous GA_3_, the NSC content in the crown buds first increased and then decreased with increasing concentrations, with the highest content at the G30 level across all three observation periods. Under exogenous IAA treatment, the NSC content showed a trend of initially increasing and then decreasing, with the highest content at the I10 level during the first observation period, significantly higher than for the control (CK) (*p* < 0.05), and at the I15 level in the subsequent two observation periods, significantly higher than for CK (*p* < 0.05). For ‘Saiwo’ alfalfa, under the treatment with exogenous GA_3_ and IAA, the NSC content in the crown buds also showed a trend of initially increasing and then decreasing with increased concentrations. The highest NSC content was at the G30 level for GA_3_ treatment across all observation periods, at the I10 level for IAA treatment during the first observation period, and at the I15 level for the subsequent two observation periods, significantly higher than for CK (*p* < 0.05).

### 2.4. Effects of Exogenous GA_3_ and IAA on Total Nitrogen Content in Alfalfa Crown Buds

**Figure 16:**
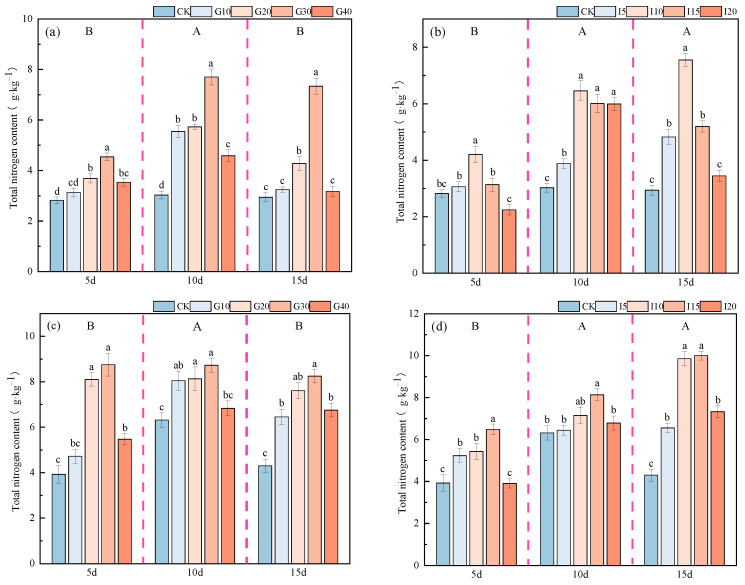
Effects of exogenous GA_3_ and IAA on total nitrogen content in the crown buds of ‘Xinmu No. 4’ (**a**,**b**) and ‘Saiwo’ (**c**,**d**) alfalfa. Different lowercase letters indicate significant differences between treatments within a certain period (*p* < 0.05); different uppercase letters indicate significant differences between different periods (*p* < 0.05).

The impact of exogenous GA_3_ and IAA on the total nitrogen content in the crown buds of ‘Xinmu No. 4’ and ‘Saiwo’ alfalfa is as shown in Figure 16. The total nitrogen content in the crown buds of both ‘Xinmu No. 4’ and ‘Saiwo’ alfalfa exhibited an initial increase followed by a decrease over time with the application of exogenous GA_3_, reaching the highest level during the second observation period, significantly higher than at other times (*p* < 0.05). With the application of exogenous IAA, the trend was an initial increase followed by stabilization, with the highest level reached during the second observation period, significantly higher than during the first observation period (*p* < 0.05). For ‘Xinmu No. 4’ alfalfa, under the treatment with exogenous GA_3_ and IAA, the total nitrogen content in the crown buds first increased and then decreased with increasing concentrations. The highest nitrogen content was observed at the G30 level for GA_3_ treatment across all three observation periods, significantly higher than for the control (CK) (*p* < 0.05). For IAA treatment, the highest nitrogen content was at the I10 level across all observation periods, also significantly higher than for CK (*p* < 0.05). For ‘Saiwo’ alfalfa, under the treatment with exogenous GA_3_ and IAA, the total nitrogen content in the crown buds showed a similar trend of initial increase followed by a decrease with increased concentrations. The highest nitrogen content was at the G30 level for GA_3_ treatment across all observation periods. For IAA treatment, the highest nitrogen content was at the I15 level across all three observation periods, significantly higher than for CK (*p* < 0.05).

### 2.5. Correlation Analysis Among Aboveground Biomass, Crown Bud Number, and Various Biochemical Parameters in Alfalfa

**Figure 17:**
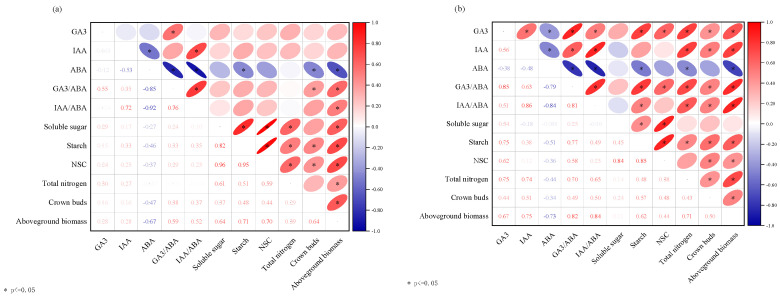
Correlation analysis between aboveground biomass and endogenous hormones after application of exogenous GA_3_ and IAA in ‘Xinmu No. 4’ (**a**) and ‘Saiwo’ (**b**) alfalfa.

As shown in Figure 17, Pearson correlation analysis was conducted to assess the relationships among endogenous hormone content, hormone ratios, soluble sugar content, starch content, NSC content, total nitrogen content, crown bud number, and biomass for ‘Xinmu No. 4’ and ‘Saiwo’ alfalfa. The results showed that biomass is positively correlated with the content of soluble sugars, starch, NSC, and total nitrogen in the crown buds. It is also positively correlated with the number of crown buds and the content of endogenous GA_3_ and IAA, while it is negatively correlated with the content of endogenous ABA. The number of crown buds in alfalfa shows a positive correlation with the content of endogenous GA_3_ and IAA. It is also positively correlated with the content of soluble sugars, starch, NSC, total nitrogen, and biomass, while it is negatively correlated with the content of endogenous ABA.

### 2.6. Regression Analysis Among Exogenous GA_3_, IAA Concentrations, and Aboveground Biomass

**Figure 18:**
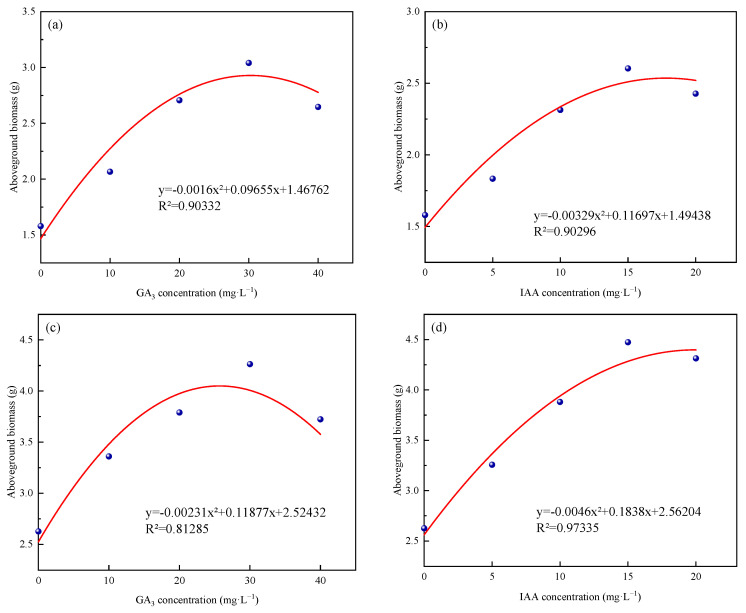
Effects of exogenous GA_3_ and IAA spraying on aboveground biomass of ‘Xinmu No. 4’ (**a**,**b**) and ‘Saiwo’ (**c**,**d**) alfalfa.

The regression analysis of different concentrations of exogenous GA_3_ and IAA with the aboveground biomass of ‘Xinmu No. 4’ and ‘Saiwo’ alfalfa is presented in Figure 18. The analysis reveals the following: According to the formula shown in (a), the aboveground biomass of ‘Xinmu No. 4’ reaches its highest at an exogenous GA_3_ concentration of 30 mg/L. As indicated in (b), the aboveground biomass of ‘Xinmu No. 4’ reaches its peak when exogenous IAA is between 15 and 20 mg/L. As shown in (c), the aboveground biomass of ‘Saiwo’ is highest at an exogenous GA_3_ concentration of 25~30 mg/L. As depicted in (d), the aboveground biomass of ‘Saiwo’ reaches its maximum at an exogenous IAA concentration of 20 mg/L.

## 3. Discussion

During the growth of plants, the content of endogenous hormones in their bodies is very low, but it plays a crucial role in regulating plant growth and development [35]. Endogenous hormones influence the normal development of plants by promoting, inhibiting, or altering their physiological processes [36]. Xie found significant differences in the response of dwarf mutant plants to exogenous IAA and GA_3_ in terms of changes in endogenous hormone levels [32]. In this experiment, the application of exogenous GA_3_ and IAA resulted in an increase in the endogenous hormone levels in the crown buds of alfalfa compared to the control (CK), with varying degrees of increase. This suggests that the external application of these hormones can significantly alter the hormonal balance within the plant. During seed germination, GA_3_ and ABA exhibit antagonistic actions. Exogenous GA_3_ can promote cell elongation and division, break dormancy in crown buds, and increase regeneration numbers [22]. Moreover, seed germination is regulated not only by endogenous hormones like GA_3_, IAA, and ABA but also by the balance between growth-promoting and growth-inhibiting hormones, especially the balance between them [37]. IAA can induce the formation of crown bud primordia, alter cell polarity transport, and promote cell differentiation to form buds, but excessively high concentrations can disrupt hormone balance and signal transduction, inhibiting crown bud regeneration. Studies have shown that exogenous gibberellin (GA) has a noticeable regulatory effect on endogenous hormones during the development of cotton bolls [38]. In this experiment, exogenous GA_3_ also exhibited significant regulatory effects on endogenous hormones in alfalfa crown buds. Ma Xueyao [39] discovered that the application of exogenous growth regulators on alfalfa at different times has varying effects on the regulation of endogenous GA_3_ content and the ratio of endogenous hormones (IAA + GA_3_ + ZR) to ABA. In this experiment, the effect of spraying different exogenous growth regulators varies depending on the regulation of endogenous GA_3_/ABA and IAA/ABA ratios in crown buds of alfalfa with different dormancy levels. After the application of exogenous GA_3_ and IAA, the ratios of GA_3_/ABA and IAA/ABA in the crown buds of “Xinmu No. 4” increased with increasing concentrations. In contrast, for “Saiwo”, the endogenous GA_3_/ABA ratio in the crown buds initially increased with concentration and then decreased. Additionally, studies indicate that GA_3_ and IAA can have synergistic or antagonistic effects, jointly regulating plant growth and development. Hormones interact with other plant hormones to form a complex regulatory network. Considering the balance and interactions among hormones is crucial for regulating plant growth and development [40,41,42]. In this experiment, the application of hormones was relatively singular, and future experiments could involve combinations of various hormones.

Exogenous growth regulators may regulate bud development by altering the distribution of sugars, amino acids, and minerals within the buds, thereby increasing the number of crown buds [43]. In this experiment, exogenous GA_3_ and IAA influenced the regeneration of alfalfa crown buds by altering the content of soluble sugars, starch, and total nitrogen, thereby increasing the number of alfalfa crown buds [44]. Higher dormancy levels correspond to a later start of the autumn dormancy period, typically featuring earlier greening, longer growth periods, higher forage yields, and faster regeneration speeds [45]. In this experiment, ‘Saiwo’ had a higher dormancy level than ‘Xinmu No. 4’ and a higher aboveground biomass per plant. However, after the application of exogenous growth regulators, ‘Xinmu No. 4’ showed a greater increase in aboveground biomass compared to the control (CK) than ‘Saiwo’, indicating that alfalfa with lower dormancy levels may achieve greater increases in aboveground biomass through the application of exogenous growth regulators than those with higher dormancy levels. Exogenous applications of GA_3_ and IAA promote crop yields [46], providing a material basis for the increase in biomass. In many studies, the potential roles of auxins and nitric oxide in the influence of humic substances on root development are linked to the presence of IAA within the supramolecular structure of humic substances. Research indicates that there is an interaction between IAA and ethylene in lateral root development, with NO also involved in auxin-induced lateral root formation [26]. Auxins have a clear promoting effect on root growth and development, and a well-developed root system is beneficial for absorbing more water and nutrients, providing sufficient resources for the growth of the aboveground parts. Gibberellins can promote the growth of the aboveground parts of alfalfa, providing a material basis for biomass accumulation. In this experiment, the increase in the number of crown buds indirectly promoted the increase in aboveground biomass. C.H. Hanson found that all environmental conditions that can increase the germination of crown buds can also improve alfalfa yield [10], and this is also confirmed in the present experiment, where the increase in the number of crown buds indirectly promoted the enhancement of aboveground biomass in alfalfa.

## 4. Materials and Methods

### 4.1. Experimental Materials

The experiment was conducted from November 2023 to June 2024 in a solar greenhouse at Xinjiang Agricultural University, with a constant temperature of 25 °C during the day and 15 °C at night, with 12 h of light and 12 h of darkness. The test materials selected were ‘Xinmu No. 4’ (Xinjiang Agricultural University) and ‘Saiwo’ (Beijing Best Grass Industry Co., Ltd., Beijing, China) alfalfa, sown in flower pots in November 2023, with 5 to 10 seeds per pot, with each variety planted in 50 pots, totaling 100 pots. The potted substrate was a 1:1 mixture of flower nutrient soil and vermiculite. After all seedlings emerged, thinning was conducted to retain 3 to 5 healthy seedlings per pot, selecting alfalfa plants of similar growth within each variety, organized into 5 groups per category, with 10 pots each.

### 4.2. Experimental Design

The experiment adopted a randomized block design. At the beginning of the flowering period of alfalfa, the plants were cut leaving a 3 cm stubble. For 3 consecutive days after cutting, different concentrations of exogenous growth regulators (without any other solvents) were sprayed on the alfalfa at 10 AM daily, with a dosage of 30 mL per pot. The experimental treatments involved two alfalfa test materials, each treated with two types of growth regulators at progressive concentrations: 10 mg/L (G10), 20 mg/L (G20), 30 mg/L (G30), 40 mg/L (G40) of GA_3_, and 5 mg/L (I5), 10 mg/L (I10), 15 mg/L (I15), and 20 mg/L (I20) of IAA. Control groups (G0, I0) consisted of alfalfa plants grown under the same conditions without treatment. Each treatment was replicated 5 times, totaling 100 pots of alfalfa plants. After the spraying treatment, samples were taken from the crown buds on the 5th day (brown buds), 10th day (brown to white buds), and 15th day (white buds) and stored at −80 °C in a Haier DW-86L419 ultra-low-temperature freezer. Samples were taken on the 15th day for analysis, with each sampling and measurement repeated 3 times to calculate the average. After regrowth to the initial flowering stage, the aboveground biomass per plant was measured using an XIUILAB FA3104 electronic scale (precision 0.0001 g, NB Chao, Foshan City, China), repeated 3 times to obtain the average value.

### 4.3. Measurement Content and Methods

(1) Determination of endogenous hormone levels in alfalfa: GA_3_, IAA, and ABA hormones were measured using ultra-performance liquid chromatography (UPLC) coupled with tandem mass spectrometry (MS/MS) [47]; the accuracy of hormone quantification ranged from 89.5% to 108.6%, with recoveries of 78.2 ± 6.5% to 92.4 ± 5.2%. The limits of detection (LOD) and quantification (LOQ) were 20–50 pg/g and 60–150 pg/g, respectively.

(2) Determination of soluble sugars and starch content: Measured using the anthrone colorimetric method [48], the method validation showed an accuracy of 90%~110% compared to certified reference materials, with spike recoveries ranging from 85% to 95%. The sensitivity was confirmed with an LOD of 5~10 μg/mL and LOQ of 10~20 μg/mL.

(3) Non-structural carbohydrates (NSC): The NSC content is the sum of soluble sugars and starch content.

(4) Total nitrogen content determination: Determined using the Kjeldahl method [49], the Kjeldahl nitrogen analyzer achieved an accuracy of 98%~102%, with spike recoveries ranging from 95% to 105%. The method sensitivity was confirmed with an LOD of 0.1 mg N and LOQ of 0.3 mg N.

### 4.4. Data Processing

All data were compiled using Microsoft Excel 2019 and analyzed using one-way ANOVA with IBM SPSS 22.0 statistical software; multiple comparisons were conducted using Duncan’s method. Graphs were plotted using Origin 2024.

## 5. Conclusions

In this experiment, the application of appropriate concentrations of exogenous GA_3_ and IAA promoted the regeneration of crown buds and increased their quantity, thereby enhancing the aboveground biomass of alfalfa. The exogenous GA_3_ treatment regulates the dynamic balance of endogenous GA_3_, IAA, and ABA in crown buds, while also affecting the distribution of nutrients such as soluble sugars, starch, and total nitrogen. The optimal application concentration of exogenous GA_3_ for promoting crown bud regeneration and increasing biomass in alfalfa was found to be 30 mg·L⁻^1^, which yielded the best results. The exogenous IAA treatment exhibited a similar regulatory pattern but demonstrated different optimal concentration effects. Exogenous treatments of GA_3_ and IAA alter the levels of endogenous hormones (GA_3_, IAA, and ABA) in alfalfa, thereby affecting carbohydrate and nitrogen metabolism and promoting crown bud regeneration. The response patterns of endogenous hormones may vary due to varietal characteristics and environmental conditions, necessitating further analysis of their molecular mechanisms for precise application. This experiment reveals the physiological mechanisms by which exogenous GA_3_ and IAA promote the regeneration of crown buds in alfalfa by regulating the endogenous hormone network and carbon–nitrogen metabolism. A functional chain of “exogenous hormones—endogenous hormones—carbon-nitrogen regulation—morphological development” has been established, enriching the theoretical system of alfalfa regeneration. This also lays the foundation for further in-depth studies on molecular mechanisms. Meanwhile, the hormone regulation strategies revealed by this study provide a reference for the cultivation management of other perennial forage grasses. In the future, exploring the optimal combinations of various hormones could further enhance the efficiency of hormone regulation in alfalfa.

## Data Availability

The data used to support the findings of this study can be made available by the corresponding author upon request.
